# Epigenetics of Chronic Stress: Mechanisms and Health Impacts

**DOI:** 10.2174/0113892029402870251108055234

**Published:** 2026-04-03

**Authors:** Kashif Abbas, Safia Habib, Mohd Mustafa, Mudassir Alam, Nazura Usmani

**Affiliations:** 1 Department of Zoology, Faculty of Life Sciences, Aligarh Muslim University, Aligarh, 202002, India;; 2 Department of Biochemistry, J.N. Medical College, Aligarh Muslim University, Aligarh, 202002, India;; 3 Department of Biological Sciences, Indian Biological Sciences and Research Institute (IBRI), Noida-201301, India

**Keywords:** Chronic stress, epigenetics, DNA methylation, non-coding RNAs, HPA axis, mental health

## Abstract

Chronic stress is a major public health concern, contributing to a spectrum of physiological and psychological disorders, including anxiety, depression, cardiovascular diseases, and metabolic syndromes. This review examines the epigenetic mechanisms mediating the impact of chronic stress on human health, focusing on DNA methylation, histone modifications, and non-coding RNAs. These epigenetic alterations act as dynamic interfaces between environmental stressors and genomic responses, modifying gene expression in critical stress-related pathways, such as the hypothalamic-pituitary-adrenal (HPA) axis and neurotrophic factors like BDNF. The significance of this research lies in elucidating how these epigenetic changes, induced by early-life adversity, socioeconomic pressures, and environmental toxins, create lasting molecular imprints that increase susceptibility to stress-related disorders and can be transmitted across generations. Key findings include stress-induced hypermethylation of the glucocorticoid receptor gene (NR3C1), histone acetylation changes in brain regions like the hippocampus and amygdala, and the regulatory roles of microRNAs (*e.g*., miR-132) and long non-coding RNAs (*e.g*., HOTAIR). These modifications disrupt neural plasticity, emotional regulation, and cognitive function, while also contributing to physical health outcomes, including inflammation, metabolic dysregulation, and cancer. The review also explores therapeutic interventions, such as DNA methylation inhibitors (*e.g*., 5-azacytidine), histone deacetylase inhibitors (HDACIs), and lifestyle modifications like mindfulness and dietary polyphenols. By integrating molecular insights with clinical perspectives, this article underscores the critical role of epigenetic research in developing targeted interventions to mitigate the enduring effects of chronic stress, offering transformative potential for personalized medicine and public health strategies.

## 1 INTRODUCTION

In the contemporary era, chronic stress has emerged as a pervasive and formidable challenge, exerting a profound influence on human health by precipitating a spectrum of physiological and psychological perturbations [1]. Stress can be defined as a sustained state of psychological or environmental pressure. Chronic stress is implicated in the aetiology of numerous disorders, including anxiety, depression, cardiovascular disease, and metabolic syndromes such as diabetes [2, 3]. Epidemiological data indicate that approximately 75–90% of primary care visits are attributable to stress-related complaints, underscoring its ubiquitous impact on morbidity [4, 5]. The physiological ramifications of chronic stress are mediated through neuroendocrine pathways such as the hypothalamic-pituitary-adrenal (HPA) axis, which governs the release of stress hormones such as cortisol and other glucocorticoids [6]. Studies have reported that prolonged activation of this axis is associated with allostatic load, a cumulative wear-and-tear on the body that predisposes individuals to disease [7, 8]. Research indicates that chronic activation of the HPA axis leads to the upregulation of pro-inflammatory factors such as interleukin 6 (IL-6) and neuropeptides like Calcitonin Gene-Related Peptide (CGRP), which contribute to lowering the threshold for headache occurrence. Evidence indicates that individuals suffering from migraines exhibit elevated levels of IL-6, interleukin-8 (IL-8), and tumour necrosis factor-alpha (TNF-α) compared to healthy controls, suggesting that these inflammatory cytokines may be involved in migraine pathogenesis [9, 10]. For example, a research study examined whether the MTHFR C677T polymorphism influences clinical and electrophysiological features of migraine by comparing genotype distributions among migraine patients with and without aura. It found that carriers of the T allele, especially those with the TT genotype, were significantly more likely to experience migraine with aura (MA) compared to individuals with the CC genotype, and they also exhibited heightened sensitivity to triggers and more frequent photophobia. Additionally, electrophysiological testing (RP-VEP) revealed distinct patterns among T-allele carriers, including reduced N75/P100 amplitudes and positive habituation indices. These findings indicate that MTHFR genotype correlates with both symptomatology and neurophysiological responses in migraine, particularly in migraine with aura [11].

Recent advancements in biomedical research have shifted the focus toward the molecular aspects of chronic stress, particularly through the view of epigenetics, which refers to the study of heritable alterations in gene expression independent of DNA sequence changes [12]. Epigenetic mechanisms such as DNA methylation, histone modifications, and the regulatory actions of non-coding RNAs (ncRNAs) serve as dynamic interfaces between environmental stimuli and genomic responses [13]. For instance, studies have demonstrated that chronic stress induces hypermethylation of the glucocorticoid receptor gene (NR3C1), attenuating its expression and impairing negative feedback regulation of the HPA axis, with methylation levels at the NR3C1 promoter region increasing by up to 20% in individuals exposed to prolonged stressors [14]. Similarly, alterations in histone acetylation and methylation patterns have been documented in stress-responsive brain regions such as the hippocampus and amygdala, correlating with a 30–50% reduction in the expression of neurotrophic factors like Brain-Derived Neurotrophic Factor (BDNF) [15, 16]. Moreover, research suggests that ncRNAs can mediate the transmission of stress-related effects across generations. For example, studies have shown that parental exposure to stress can alter ncRNA expression in gametes, leading to changes in offspring phenotype [17, 18]. This can result in behavioral abnormalities or other health issues in subsequent generations, even without direct exposure to the original stressor.

The sources of chronic stress are multifaceted, encompassing socioeconomic pressures [19], discrimination, traumatic experiences, and environmental adversities, each contributing to a cascade of molecular events that reshape the epigenome [20]. Upon cortisol binding, glucocorticoid receptors (GRs) undergo conformational activation and translocate into the nucleus, where they interact with glucocorticoid response elements (GREs) within target gene promoters and enhancers. This interaction facilitates the recruitment of epigenetic modifiers, including DNA methyltransferases and histone-modifying enzymes, leading to alterations in DNA methylation patterns and histone post-translational modifications [21]. These epigenetic modifications are not transient; they can persist across generations *via* transgenerational epigenetic inheritance, as evidenced by animal models where offspring of stressed progenitors exhibit altered DNA methylation profiles at stress-related loci, increasing disease susceptibility by 15–25% [22]. Stress can lead to changes in histone acetylation and methylation. For instance, chronic stress or drug exposure alters HDAC5 activity in reward-related brain regions (*e.g*., nucleus accumbens), affecting histone acetylation and gene expression [23]. Moreover, it has been studied that, persistent stress can contribute to insomnia through a variety of mechanisms, including the activation of the hypothalamic-pituitary-adrenal (HPA) axis, alterations in brain chemistry, and disruptions to arousal systems. These physiological and psychological changes can make it difficult to fall asleep and stay asleep, creating a cycle of stress and sleeplessness [24, 25]. For instance, stress induced HPA axis activation triggers the release of stress hormones cortisol, which interfere with Rapid Eye Movement (REM) and slow-wave sleep, both crucial for restorative sleep [26]. Furthermore, Stress can impact the levels of neurotransmitters and receptors in the brain involved in sleep regulation. For example, stress can affect the expression of glucocorticoid receptors (GR) and mineralocorticoid receptors (MR) on brain astrocytes, which are important for regulating sleep. It can also disrupt arousal systems like the noradrenergic locus coeruleus, leading to increased wakefulness and difficulty initiating sleep [27, 28].

This study aims to elucidate the epigenetic foundations of chronic stress, dissecting the roles of DNA methylation, histone modifications, and non-coding RNAs in modulating stress responses. It further explores the downstream effects on brain function, particularly in regions critical for memory and emotion regulation and the broader implications for physical health, including cardiovascular, metabolic, and immune systems. By integrating molecular insights with clinical perspectives, this review seeks to explore potential therapeutic ways to ease the persistent consequences of stress on human health.

## 2 EPIGENETICS AND STRESS: A CONCEPTUAL FRAMEWORK

The intersection of epigenetics and the complex environment of chronic stress creates a broad and in-depth conceptual framework, backed by substantial molecular and genetic research findings. Epigenetics, which examines heritable modifications in gene expression without altering the DNA sequence, functions as a regulatory mechanism for the body's response to stress. DNA methylation is an essential epigenetic process that incorporates methyl groups onto cytosine bases, mainly occurring at CpG dinucleotides. It influences the repression or expression of genes [29, 30]. Molecular research has shown that DNA methylation patterns are dynamic under the effect of prolonged stress. For instance, studies have shown substantial stress-induced DNA methylation changes in important stress response genes such as the glucocorticoid receptor gene (NR3C1) and Brain-Derived Neurotrophic Factor (BDNF) [15, 31, 32]. Moreover, the study by Hing *et al.* (2018) explored how chronic social defeat stress alters DNA methylation patterns in the dentate gyrus of mice using Methyl-Seq and bisulfite-pyrosequencing. They identified two differentially methylated regions (DMRs), one within intron 9 of Drosha showing reduced methylation, and another on chromosome X with increased methylation in stressed mice. The chromosome X DMR replicated consistently across independent mouse cohorts and in postmortem brains from individuals with major depressive disorder (MDD). These findings reveal novel stress-associated methylation changes that may contribute to the molecular mechanisms underlying MDD [33]. These epigenetic changes have been linked to differences in stress sensitivity and resistance, giving direct empirical proof of the complex genetic-epigenetic interactions that shape stress responses. Furthermore, histone modifications, which finely modify chromatin architecture, have been identified as key participants in gene accessibility. Not only has genetic research proved the importance of genetic differences in interacting with histone modification patterns, but it has also identified particular genes and loci that are sensitive to histone modifications [34]. These findings support the complex genetic-epigenetic interactions that underpin individualised stress responses. Notably, genes producing hypothalamic-pituitary-adrenal (HPA) axis components, such as NR3C1 and the Corticotropin-Releasing Hormone (CRH), demonstrate histone modifications under chronic stress circumstances [35, 36]. Specific genetic variations within NR3C1, such as the BclI polymorphism (rs41423247) and N363S polymorphism (rs6195), have been discovered to interact with histone changes at key regulatory areas, including enhancers, promoters, and other cis-regulatory elements. These interactions influence the stress response and vulnerability to stress-related diseases and disorders [37]. In addition to these findings, non-coding RNAs (ncRNAs), which include microRNAs (miRNAs) and long non-coding RNAs (lncRNAs), act as key regulators of post-transcriptional gene expression, demonstrating their importance in defining the molecular stress response [38, 39]. Specific miRNAs and lncRNAs, such as miR-132 and BDNF-AS, have been identified as playing critical roles in the molecular response to chronic stress [40, 41]. The genetic variances in the genomic areas encoding stress-responsive ncRNAs, as well as the reciprocal effect of these genetic variations on ncRNA expression patterns, have been discovered *via* research [42]. These results establish a complicated network of genetic-epigenetic-RNA connections that drive chronic stress response.

Table **1** provides a broad overview of the epigenetic mechanisms (DNA methylation, histone modifications, and non-coding RNAs) and their interactions with stress-related genes and pathways, such as the glucocorticoid receptor gene (NR3C1), BDNF, and HPA axis components.

## 3 THE RELATIONSHIP BETWEEN ENVIRONMENTAL FACTORS AND EPIGENETIC MODIFICATIONS

Early life events may have a profound influence on epigenetic regulation [43]. Early adversity, such as prenatal stress or childhood abuse, can cause long-lasting epigenetic alterations [44]. Research findings show that these experiences are often relatedto changes in DNA methylation at crucial loci, such as the NR3C1 [45]. In this context, the interplay between genetic and epigenetic factors plays a fundamental role in modulating an individual's stress resilience and influencing their susceptibility to stress-related disorders later in life. Nutrition has a critical role in coordinating epigenetic controls, according to a substantial study [46-48]. Folate and other methyl donors are critical nutrients for DNA methylation processes [49]. On the other hand, nutrient deficient or fat rich diets cause abnormal epigenetic alterations [50]. For instance, several studies confirmed that, Vitamins and mineral deficiencies, especially vitamins B and omega-3 fatty acids, can disrupt epigenetic processes, which affect gene expression related to metabolism, inflammation, and other cellular functions [51, 52]. Genes critical to metabolic pathways, inflammation, and brain function, including, PPARγ [53], SIRT1 [54], FTO [55], TNF-α, IL-6, NF-κB [56], BDNF, CREB, and COMT [57], have been identified as susceptible to epigenetic modifications in response to dietary variables. Genetic variables closely connect with these epigenetic dietary impacts, emphasising the complexity of the interaction. Research suggests that the dietary choices of expectant mothers during pregnancy can lead to enduring epigenetic alterations in the foetus [58]. Notably, these changes affect genes involved in crucial developmental pathways such as insulin-like growth factor 2 (IGF2), PPARγ [59], glucocorticoid receptor (GR), and BDNF [60]. Folate deficiency during pregnancy can induce DNA methylation alterations at genes crucial for neural tube development, including methylenetetrahydrofolate reductase (MTHFR) [61], neural tube defect gene 1 (NTD1) [62], and LRP6 (Low-density lipoprotein receptor-related protein 6), among others [63]. Furthermore, genetic alterations within the stress response system (SRS), such as polymorphisms in the MTHFR gene, can increase an individual’s vulnerability to stress-related headache disorders, highlighting the influence of individual genetic makeup in the underlying pathophysiology [64]. Environmental pollutants create a permanent epigenetic imprint that is strengthened by genetic factors. For instance, DNA methylation changes in genes related to respiratory health are observably caused by airborne pollution. An individual's sensitivity to the negative effects of environmental exposures is ultimately determined by this process, which is marked by a genetic-epigenetic interaction [65, 66]. Research has shown that exposure to environmental contaminants, such as polycyclic aromatic hydrocarbons (PAHs), alters the DNA methylation of genes related to lung growth and development, including genes like CC16 [67], SFTPB [68], and FOXF1 [69]. The epigenetic response to PAH exposure may be further influenced by genetic variations in key metabolic and respiratory health-related genes, such as those encoding enzymes involved in PAH metabolism such as CYP1A1, CYP1B1, GSTM1, GSTT1, NAT1, NAT2 [70], surfactant proteins such as SFTPA1, SFTPB, SFTPC, SFTPD, and beta-adrenergic receptors (ADRB1, ADRB2, ADRB3), potentially impacting an individual's susceptibility to respiratory illnesses [71]. Individual lifestyle decisions, such as drug addiction and tobacco use, have a complex relationship with the epigenetic landscape. CYP1A1, controlling detoxification pathway target of epigenetic alterations as a result of these behaviours [72, 73]. In addition, genetic variables also complexly regulate the degree and kind of epigenetic changes brought about by lifestyle decisions. Study shows smoking tobacco causes alterations in DNA methylation at genes linked to cardiovascular health, such as endothelial nitric oxide synthase (eNOS) [74], and lung cancer (p16INK4a) [75]. Variants in these genes may affect an individual's vulnerability to smoking-related disorders by altering the epigenetic response to smoking. Fig. **1** depicts a schematic representation of the interplay between environmental factors and epigenetic modifications which leads to a persistent stress condition.

## 4 DNA METHYLATION DYNAMICS: AN INSIGHT INTO STRESS-INDUCED CHANGES AND IMPLICATIONS

DNA methylation is a fundamental epigenetic process extensively studied in the context of chronic stress, playing a critical role in shaping an individual’s resilience and stress response. This process involves the addition of methyl groups to cytosine bases, primarily at CpG dinucleotides, influencing gene expression by either repressing or activating transcription [29]. Stress-responsive genes undergo significant changes in DNA methylation patterns under prolonged stress, with both hypermethylation and demethylation contributing to dysregulation depending on the gene and context. For instance, early-life stress is associated with hypermethylation at the promoter region of the glucocorticoid receptor gene (NR3C1), reducing its expression and impairing glucocorticoid-mediated feedback regulation of the hypothalamic-pituitary-adrenal (HPA) axis. This hypermethylation, observed to increase methylation levels by up to 20% in stressed individuals, is linked to heightened stress reactivity and increased susceptibility to mental health disorders such as depression and post-traumatic stress disorder (PTSD) [76-78]. Similarly, stress-induced hypermethylation of the Brain-Derived Neurotrophic Factor (BDNF) gene reduces its expression, disrupting neural plasticity and contributing to cognitive deficits and mood disorders [79, 80].

However, the generalization that increased methylation (hypermethylation) is universally detrimental and demethylation is beneficial oversimplifies the complex role of DNA methylation in stress responses. Demethylation of specific genes under chronic stress can also lead to dysregulation with adverse consequences. For example, the FKBP5 gene, a glucocorticoid receptor co-chaperone critical for stress resilience, exhibits stress-induced demethylation at specific CpG sites in its promoter region, leading to increased gene expression. This overexpression disrupts HPA axis regulation, enhancing glucocorticoid signalling and increasing vulnerability to stress-related disorders such as depression and PTSD [81]. Remarkably, Klengel *et al.* (2013) demonstrated that a functional polymorphism in FKBP5 interacts with childhood trauma to induce allele-specific DNA demethylation in glucocorticoid response elements, resulting in enhanced FKBP5 transcription. This demethylation leads to long-term dysregulation of the stress hormone system, altered immune cell function, and changes in brain regions associated with stress regulation, thereby increasing the risk of psychiatric disorders in adulthood [82]. Similarly, stress-induced demethylation of immediate early genes like c-Fos and EGR1 in the hippocampus and amygdala enhances their expression, contributing to heightened neuronal activity and emotional reactivity. These changes are associated with impaired memory consolidation and increased risk of mood disorders, as excessive activation of these genes disrupts synaptic plasticity and emotional regulation [83, 84]. These examples highlight that demethylation, like hypermethylation, can have maladaptive effects, depending on the gene and its role in stress-related pathways.

The broader implications of stress-induced DNA methylation changes, whether hypermethylation or demethylation, extend beyond immediate stress responses to long-term mental and physical health outcomes. For instance, hypermethylation at NR3C1 and BDNF loci is linked to chronic inflammation and metabolic dysregulation, while demethylation of FKBP5, c-Fos, and EGR1 can exacerbate psychiatric vulnerabilities and cognitive impairments [81, 85]. Moreover, these epigenetic modifications can have generational effects, as demonstrated by studies showing that ancestral stress exposure alters DNA methylation patterns in offspring, increasing disease susceptibility several-fold [86, 87]. These findings underscore the dynamic and context-dependent nature of DNA methylation, where both increased and decreased methylation can contribute to stress-related pathologies. Understanding these nuances opens avenues for targeted therapeutic interventions, such as DNA methylation inhibitors (*e.g*., 5-azacytidine) or demethylation-modulating therapies, to restore balanced gene expression and mitigate the adverse effects of chronic stress on health [88].

## 5 HISTONE MODIFICATIONS: ROLE IN REGULATING THE STRESS RESPONSE

The fact that DNA methylation is a dynamic process in response to stress emphasises how important this epigenetic imprint is in determining how resilient or susceptible a person is to stress. These results provide insights into possible treatment paths wherein DNA methylation-targeting therapies may lessen the negative effects of stress and enhance mental health. To fine-tune the stress response, these modifications in histone acetylation levels interact with genetic differences, such as polymorphisms in the NR3C1 [89], thereby influencing an individual's physiological and behavioural responses to stressors. The complex process of histone methylation may either promote or inhibit the expression of a gene, depending on which particular histone and residue gets methylated [90]. The amino acid residues primarily involved in histone methylation are lysine and arginine [91]. Methylation can occur on specific lysine residues, such as lysine 4, 9, 27, and 36 on histone H3, and lysine 20 on histone H4 [92]. Arginine residues on histones, particularly arginine 3, 8, and 17 on histone H3, can also undergo methylation [93]. Depending on which of these residues is methylated and to what extent, histone methylation can either promote or inhibit the expression of a gene by modulating chromatin structure and recruitment of transcriptional regulators. For instance, although methylation at H3K9 and H3K27 is normally repressive [94], methylation of histone H3 at lysine 4 (H3K4me3) is linked to gene activation [95]. Studies have shown the special interaction between histone methylation and stress control. Stress-responsive genes show specific patterns of histone methylation while under stress such as the BDNF gene. Here, variations in BDNF expression have been connected to modifications in H3K4me3 and H3K27me3, which in turn affect neural plasticity and stress adaptation [96-98].

Histone changes interact with non-coding RNAs and DNA methylation rather than acting alone [99]. The vulnerability and stress response are fine-tuned by this complex interaction. Studies have indicated that the stress-responsive microRNA miR-132 modifies chromatin accessibility at stress-responsive loci by influencing the expression of specific histone acetyltransferases, such as CBP, p300, and PCAF, as well as histone deacetylases, including HDAC1 and HDAC2 [100-102]. Schematic representation of histone modifications andtheir role in regulating the stress responseis depicted in Fig. **2**.

## 6 NON-CODING RNAs: MicroRNAs AND LONG NON-CODING RNAs IN STRESS-RELATED EPIGENETIC CHANGES

MiRNAs, which are short RNA molecules with an average length of 20–22 nucleotides, are essential for the regulation of genes post-transcriptionally [103]. These molecules become mature miRNAs by going through several processing stages from longer original miRNA transcripts (pri-miRNAs). When mature miRNAs interact with target mRNAs' 3' untranslated region (3'UTR), translational suppression or mRNA destruction results [104]. MiR-132 has become a key figure in the epigenetic regulation of genes that respond to stress. miRNAs such as miR-124 regulate stress resilience by targeting key genes like HDAC4, HDAC5, GSK3β, GR, and BDNF. In stressed mice, decreased miR-124 correlates with depressive-like behaviors, while overexpression confers resilience [105]. By focusing on histone acetyltransferases and deacetylases, this miRNA affects chromatin accessibility [106, 107]. This particular regulatory network is an illustration of how histone modifications and miRNAs interact dynamically. Research is now being done to determine how genetic variants affect interactions between miRNAs. Under stressful conditions, the regulatory networks may be adjusted by variations in the miRNA genes or in the miRNA-binding sites [108]. To modify stress sensitivity, genetic variations in the BDNF gene, a crucial component of neural plasticity, may interact with miRNA-mediated control [109]. Stress-induced epigenetic changes are mediated by a diverse array of RNA molecules known as long non-coding RNAs (lncRNAs), which are a heterogeneous collection of molecules longer than 200 nucleotides [110]. Theyare involved in a wide range of processes, including post-transcriptional processing, transcriptional control, and chromatin remodelling [111]. Key regulators of stress reactions have been identified as certain lncRNAs. Studies show altered DNA methylation patterns at stress-responsive genes that have been linked to the lncRNA HOTAIR (HOX transcript antisense RNA) [112, 113]. The interaction between miRNAs and lncRNAs is dynamic. The miRNAs are sequestered away from their mRNA targets by lncRNAs functioning as competitive endogenous RNAs (ceRNAs) [114, 115].

## 7 CHRONIC STRESS AND BRAIN EPIGENETICS

Long-term stress induces long-lasting epigenetic modifications in the brain at the molecular level, which mostly takes the form of altered DNA methylation. Especially, NR3C1 serves as an example of how these alterations are most noticeably shown at genes controlling the stress response. Numerous studies have shown that the promoter region of NR3C1 exhibits hypermethylation patterns in response to prolonged stress [116, 117]. Wide-ranging effects result from this epigenetic modification, which makes people less able to regulate their levels of glucocorticoids effectively and exacerbates the stress response [118]. Study shows that changes in DNA methylation at BDNF loci in this environment affect the production of the protein, which in turn affects brain plasticity and cognitive performance [119]. Changes in DNA methylation at BDNF loci influence the production of several proteins crucial for brain function. BDNF supports neuronal survival, differentiation, and synaptic plasticity. Methyl-CpG-binding protein 2 (MeCP2) binds to methylated DNA, affecting gene expression and neuronal development [120]. Tropomyosin receptor kinase B (TrkB) acts as the receptor for BDNF, mediating its effects on neuronal function and plasticity [121]. DNA methyltransferases (DNMTs) are enzymes responsible for adding methyl groups to DNA. Changes in their expression or activity can impact DNA methylation patterns, including those at BDNF regulatory regions [122]. Histone deacetylases (HDACs) remove acetyl groups from histone proteins, influencing chromatin structure and gene expression [123]. Dysregulation of HDAC activity can affect the expression of genes like BDNF through epigenetic mechanisms [124]. cAMP response element-binding protein (CREB) is a transcription factor involved in synaptic plasticity and memory formation. BDNF signalling through TrkB receptors can activate CREB, leading to the transcription of genes involved in synaptic plasticity [125]. Activity-regulated cytoskeleton-associated gene (Arc) is an immediate early gene involved in synaptic plasticity and memory consolidation. These proteins collectively play crucial roles in shaping brain plasticity and cognitive performance [126]. Changes in the histone acetylation and methylation patterns of stress-responsive genes, including the CRH gene [127], serve as an instance of the phenomenon. Studies have shown that the continuous stimulation of the HPA axis in the context of chronic stress results in histone changes that influence the expression of CRH, thereby sustaining the stress response [128, 129]. Chronic stress's epigenetic effects are mostly mediated by non-coding RNAs (ncRNAs), especially lncRNAs and miRNAs. Chronic stresses cause distinct miRNA profiles to appear, which change the brain's gene expression patterns [130]. LncRNAs also affect gene expression and chromatin structure to achieve their desired effects. An altered DNA methylation pattern at stress-responsive genes has been linked to the lncRNA HOTAIR. These non-coding RNAs have a critical role as mediators, regulating the brain's epigenetic response to long-term stress [131, 132].

Being essential for both memory consolidation and cognitive function, the hippocampus is especially vulnerable to the long-term epigenetic consequences of chronic stress [133]. Moreover, the hippocampus is a dynamic epigenetic regulator of genes like c-Fos and Arc, which are essential for synaptic plasticity and long-term potentiation (LTP) [134, 135]. At the promoter region of genes like c-Fos and Arc, alterations in DNA methylation and histone acetylation patterns occur, directly impacting the accessibility of these promoters to transcriptional machinery and subsequently influencing the expression levels of c-Fos and Arc. Dysregulation of these epigenetic modifications at the promoters of c-Fos and Arc genes has been linked to cognitive deficiencies associated with prolonged exposure to stress [136, 137]. Changes in DNA methylation at stress-responsive genes inside the amygdala, such as the CRH gene, maintain amygdala hyperactivity [138]. Elevated amygdala response to stress is linked to higher emotional reactivity and the development of mood disorders [139]. Furthermore, genes associated with emotional memory control, such as the Early Growth Response 1 (EGR1) gene, as well as FosB, NR4A, and CREB, display epigenetic modifications in the amygdala [140]. The cognitive disadvantages caused by stress-induced alterations in the hippocampus highlight the difficulties people may experience while developing and retrieving memories. These changes might lead to cognitive deficits seen in diseases like post-traumatic stress disorder (PTSD) [141, 142]. Aforementioned factors have been linked to the development of mood disorders such as sadness and anxiety [143, 144]. Fig. **3** illustrates interplay between chronic stress and epigenetic modification in the brain.

## 8 CHRONIC STRESS AND ITS ROLE IN MODIFYING EPIGENETIC MARKERS ASSOCIATED WITH PHYSICAL HEALTH OUTCOMES

Chronic stress sets off a chain of epigenetic changes that may have a major influence on physical health consequences (Fig. **4**). DNA methylation is one significant process by which this happens. Changes in DNA methylation patterns caused by stress have been reported at genes involved in inflammation, immunological function, and metabolic control. Epigenetic modification of genes involved in the pro-inflammatory response, such as Tumour Necrosis Factor (TNF) and Interleukin-6 (IL-6), may result in an inflammatory state being exacerbated [145]. Chronic low-grade inflammation is a major contributor to a variety of chronic illnesses, including cardiovascular disease and diabetes [146]. Chronic stress-induced epigenetic changes, namely DNA methylation, have been related to altered gene expression patterns associated with cancer risk. Tumour suppressors and DNA repair genes are examples of genes whose hypermethylation may enhance carcinogenesis by inhibiting genes essential for preserving genomic integrity [147]. Study shows that tumour suppressor p53 gene (TP53) may become hypermethylated, which may interfere with its normal function in DNA repair and cell cycle control and raise the risk of cancer [148-150]. Analogously, variations in methylation at DNA repair genes, such as BRCA1, may impair the response to DNA damage and raise the risk of developing breast and ovarian cancer [151, 152]. Changes in insulin sensitivity and secretion are linked to modifications in DNA methylation at the insulin gene (INS), which in turn affects the consequences of metabolic health [153, 154]. The relationship between long-term stress and physical health consequences is further supported by histone alterations, which are often thought of as dynamic regulators of gene expression. Tumorigenesis may be aided by the silence of tumour suppressor genes as a result of altered histone modifications [155]. Retinoblastoma 1 (RB1) gene is one of the genes where histone deacetylation may cause uncontrollably fast cell cycle progression, raising the risk of retinoblastoma and other malignancies [156]. Changes in histone acetylation and methylation patterns at genes involved in immune response and metabolism control may result from long-term stress [157]. For instance, modifications in appetite control and obesity have been linked to variations in histone acetylation patterns at the Leptin gene (LEP) [158]. Histone changes at blood pressure-regulating genes, including the Endothelin-1 gene (EDN1), have been connected to the onset of hypertension in the setting of cardiovascular health [159]. Therefore, the cumulative effect of histone modifications may be linked to increased cardiovascular risk factors and diseases. Stress-responsive miRNAs specifically target genes related to metabolism, immunological control, and cellular homeostasis. Several cancer types have been linked to enhanced cell proliferation, invasion, and reduced apoptosis in response to the well-known stress-responsive miRNA miR-21 [160]. Another stress-responsive miRNA, miR-34a, has also been associated with a higher risk of colorectal cancer and other cancers *via* its downregulation [161]. Changes in miRNA profiles may have an effect on immune responses and the balance of metabolism, which may result in chronic inflammation and metabolic syndrome. Stress-responsive miRNA that can target genes linked to immune function is miR-155, which may help to create a pro-inflammatory state [162, 163].

## 9 EPIGENETICS, STRESS, AND MENTAL HEALTH

There is strong evidence linking DNA methylation, chronic stress, and mental health issues (Fig. **5**). Research into DNA methylation has highlighted its role not only in stress-related psychiatric disorders but also in resilience and adaptation. For instance, FKBP5, a gene encoding a glucocorticoid receptor co-chaperone, which has been implicated in stress resilience [164], and the OXTR gene, encoding the oxytocin receptor, which has been associated with social support buffering against stress [165]. Moreover, recent advances in epigenome-wide association studies (EWAS) have identified novel candidate genes and pathways associated with stress-related phenotypes, providing a comprehensive understanding of the epigenetic landscape underlying psychiatric disorders. Studies have highlighted genes such as Serotonin Transporter (SLC6A4), Catechol-O-Methyltransferase (COMT), and Corticotropin-Releasing Hormone Receptor (CRHR1), as well as pathways including neurotrophin signalling and inflammatory pathways, as key players in stress response regulation and mental health outcomes [166-168]. Histone modifications, including acetylation, methylation, phosphorylation, and ubiquitination, collectively orchestrate chromatin remodelling and gene expression. Beyond histone acetylation, studies have implicated other histone modifications in the aetiology of mental health disorders. For instance, alterations in histone methylation patterns, particularly at the H3K4 (histone H3 lysine 4) and H3K27 (histone H3 lysine 27) sites, have been associated with depression and anxiety disorders [169-171]. Specific genes involved in these histone modifications include those encoding histone methyltransferases such as EZH2, SUV39H1 [172], and demethylases like LSD1, JARID1A, which regulate the addition and removal of methyl groups on histone tails, respectively [173]. Dysregulation of miRNAs has emerged as a significant contributing factor to the pathophysiology of conditions like depression and anxiety. For instance, levels of circulating miR-663 and miR-4505 have been found to correlate with anxiety symptoms [174], while miR-29c expression has been observed to increase in response to social anxiety during a social stress task [175]. This upregulation corresponds not only to the stress experience itself but also to changes in ventromedial prefrontal cortex functional connectivity. Adding to the complexity, single-nucleotide polymorphisms (SNPs) occurring in the precursors or mature microRNAs have the potential to disrupt balanced processing in anxiety. Research suggests that SNPs linked to specific microRNA candidates have revealed numerous microRNA variations, including miR-22, miR-138-2, miR-148a, miR-339, and miR-488, which have associations with anxiety-related disorders such as panic disorder [176, 177]. These investigations have revealed abnormal microRNA expression and potential dysregulate (in anxiety, providing valuable insights into the role of microRNAs in anxiety-related disorders.

## 10 INTERVENTIONS AND POTENTIAL THERAPIES

5-azacytidine and decitabine, two DNA methylation inhibitors (DMIs) [178], can reverse abnormal DNA methylation patterns [179]. Table **2** summarises various DMIs with their mode of action. These inhibitors might be used to restore normal methylation patterns at important genes in the setting of stress-related health disorders [180], thereby alleviating the effect of chronic stress on illnesses such as depression and anxiety. Histone deacetylase inhibitors (HDACIs) may affect gene expression by modifying histone acetylation patterns. HDACIs may be investigated in the context of stress as a strategy to normalise abnormal histone acetylation patterns, possibly giving therapeutic paths for mood disorders and cognitive deficiencies associated with chronic stress [181, 182]. Stress-reduction strategies such as mindfulness meditation and yoga may alter epigenetic changes linked to chronic stress [183]. These approaches may result in less DNA methylation at stress-related genes, which may contribute to enhanced mental and physical well-being [184, 185]. Diet and nutrition are critical in modifying epigenetic markers. Polyphenols found in green tea and resveratrol found in red wine have shown promise in affecting DNA methylation patterns [186, 187]. Precision medicine, an emerging subject, shows enormous potential in personalising therapies to each individual's unique epigenetic profile. Personalised therapy techniques may be devised by characterising a person's individual epigenetic changes, targeting their specific vulnerability to stress-related health conditions [188, 189].

## CONCLUSION

Chronic stress has emerged as a critical mediator of disease through its ability to induce persistent epigenetic modifications that dysregulate essential physiological and neurological processes. Research has firmly established that stress-induced alterations in DNA methylation (such as hypermethylation of the glucocorticoid receptor gene NR3C1), histone modifications (including altered acetylation patterns in stress-responsive brain regions like the hippocampus and amygdala), and non-coding RNA activity (*e.g*., miR-132 and HOTAIR) contribute significantly to maladaptive stress responses. These epigenetic changes impair neuroplasticity, disrupt the hypothalamic-pituitary-adrenal (HPA) axis, and promote systemic inflammation, thereby increasing susceptibility to a spectrum of disorders ranging from anxiety, depression, and PTSD to cardiovascular disease, metabolic syndrome, and even cancer. These epigenetic modifications are not static, they can be influenced by early-life adversity, socioeconomic factors, and environmental exposures, creating a lasting biological imprint that may even be transmitted to subsequent generations. This transgenerational epigenetic inheritance underscores the profound and far-reaching consequences of chronic stress, suggesting that its effects extend beyond the individual to shape population health trends. The dynamic nature of epigenetic regulation offers promising opportunities for intervention. Pharmacological approaches such as DNA methyltransferase inhibitors (*e.g*., 5-azacytidine) and histone deacetylase inhibitors (HDACIs), have shown potential in preclinical models to reverse stress-induced epigenetic changes. However, their clinical application requires further refinement to improve specificity and reduce off-target effects. Beyond pharmaceuticals, lifestyle and behavioral interventions such as mindfulness-based stress reduction (MBSR), physical exercise, and dietary modifications rich in polyphenols and omega-3 fatty acids have demonstrated epigenetic-modulating effects. These non-invasive strategies not only enhance stress resilience but also promote broader health benefits, making them viable options for long-term management. To fully realize the therapeutic potential of epigenetic interventions, future research must focus on several critical areas. First, the discovery of reliable epigenetic biomarkers for chronic stress exposure is essential to enable early detection and facilitate personalized treatment strategies. Second, researchers need to achieve greater mechanistic clarity by elucidating the precise interactions between different epigenetic modifications, particularly the cross-talk between DNA methylation and miRNA regulation within stress-related pathways. Third, comprehensive transgenerational studies are needed to investigate how stress-induced epigenetic changes are inherited across generations and explore potential interventions to mitigate these effects in offspring. Finally, expanded clinical trials in human populations, especially among high-risk groups such as individuals with childhood trauma or chronic illness, will be crucial for validating the efficacy and safety of emerging epigenetic therapies. Together, these research priorities will advance our ability to translate epigenetic discoveries into effective clinical applications for stress-related disorders. The growing understanding of stress-related epigenetics bridges the gap between environmental exposures and biological vulnerability, offering transformative insights into disease prevention and treatment. By integrating molecular discoveries with clinical and public health strategies, we can develop targeted interventions to enhance stress resilience, disrupt the cycle of intergenerational risk, and ultimately improve both mental and physical health outcomes. The next decade of research holds immense promise for translating epigenetic knowledge into actionable solutions, paving the way for a more proactive and personalized approach to combating the pervasive effects of chronic stress.

## Figures and Tables

**Fig. (1):**
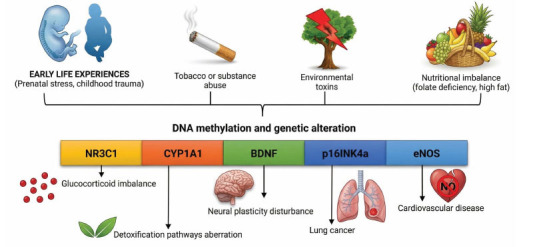
Various environmental and lifestyle factors such as early life experiences, substance abuse, environmental toxins and nutritional imbalance act as stressors for body. Stress response occurs in the form of epigenetic modifications such as DNA methylation or genetic alteration. NR3C1 methylation leads to an imbalance in glucocorticoidlevels on the other hand methylation of CYP1A1 leads to disturbance in detoxification pathways. Cardiovascular diseases are caused by methylation of genes for eNOS, while epigenetic modification in p16INK4a leads to the onset of lung cancer.

**Fig. (2):**
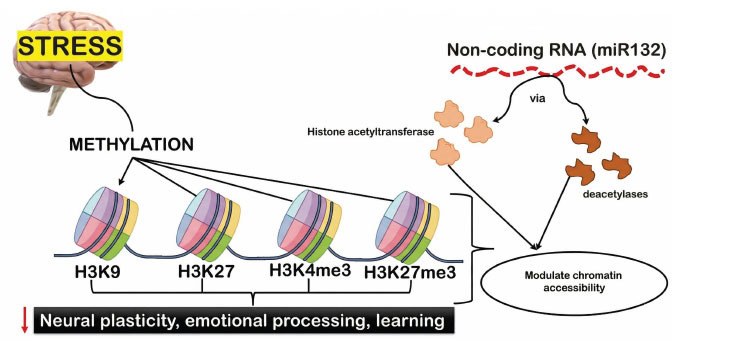
Stress leads tomodifications in histone. Preferredsites for histone methylation are H3K9, H3K27, H3K4me3, and H3K27me3, which hamper several physiological activities such as neural plasticity, emotional processing and learning. Furthermore, stress induces the role of non-coding RNA, namely miR132, which modulate chromatin accessibility *via* regulating activities of histone acetyltransferases and deacetylases.

**Fig. (3):**
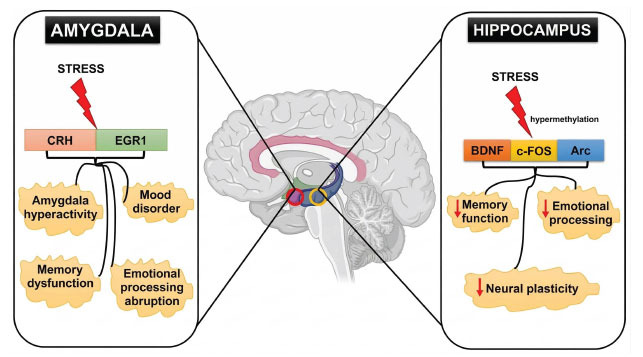
Chronic stress plays key role in epigenetic modification of brain. Two important part of brain limbic system that is amygdala and hippocampus are involved in it. Under chronic stress condition, Corticotropin Releasing Hormone (CRH) and Early growth response gene-1 (EGR1) gene in amygdala undergo epigenetic modification which causes hyperactivity of region leading to mood disorder, memory dysfunction and difficulty in emotional processing. On the other hand, under stress condition, in hippocampal region, gene namely Brain derived neurotrophic factor (BDNF), c-FOS and Arc undergo epigenetic modification which leads to problem such memory dysfunction, low neural plasticity and difficulty in emotional processing.

**Fig. (4):**
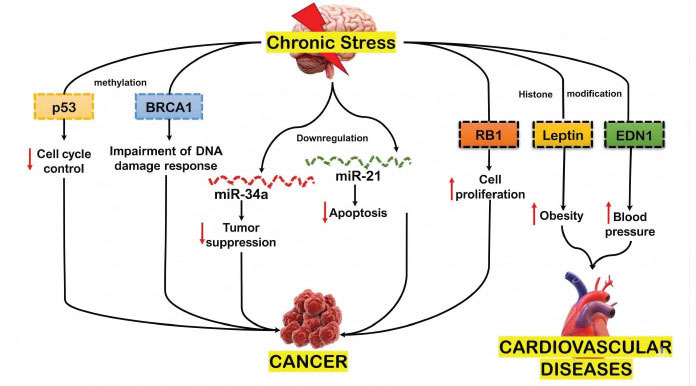
Chronic stress poses detrimental impact on physical health *via* epigenetic modification. It could lead to various physiological ailments and diseases such as cancer, obesity, hypertension and cardiovascular disease. Under chronic stress, genes such as p53 and BRCA1 under methylation which leads to loss of cell cycle control and impairment of DNA repair machinery respectively. Furthermore, under chronic stress condition, miR-34a and miR-21 are downregulated which leads to failure of tumour suppression machinery and loss of apoptosis respectively. It is also observed that chronic stress leads to histone modification which further leads to dysregulation of certain genes. RB1 gene dysregulation leads to increased cell proliferation which contribute to cancer onset. Dysregulation of leptin gene causes health related issues such as obesity. EDN1 gene regulates vasoconstriction of blood vessels. Dysregulation of EDN1 gene due to epigenetic modification can lead to hypertension and various cardiovascular complications.

**Fig. (5):**
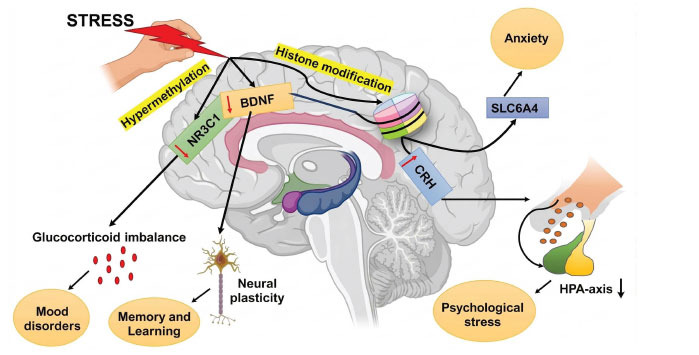
Chronic stress hurts mental health. Under stress conditions, the genes which regulate mental well-being are dysregulated. It is observed that hypermethylation of NR3C1 causes glucocorticoid imbalance, which is associated with mood disorders, while methylation of BDNF gene disturbs neural plasticity, which hampers memory and learning. Furthermore, histone modification has been shown to dysregulate of genes such as CRH and SLC6A4. Dysregulation of the CRH gene disturbs functioning of the HPA-axis which further leads to psychological stress while SLC6A4 abruption leads to onset of anxiety disorder.

**Table 1:** Key genes, pathways, and epigenetic regulators associated with chronic stress.

**Gene**	**Pathway/Function**	**Epigenetic Regulator**	**Impact of Epigenetic Modification**
NR3C1	HPA axis regulation, glucocorticoid receptor function	DNA methylation (hypermethylation at promoter), histone acetylation, miR-132	Impaired glucocorticoid feedback, increased stress reactivity, and mood disorders
BDNF	Neural plasticity, synaptic function, cognitive performance	DNA methylation, histone methylation (H3K4me3, H3K27me3), miR-132, miR-26a, miR-26b	Reduced expression, disrupted neural plasticity, cognitive deficits, and depression
CRH	HPA axis activation, stress response	Histone acetylation, methylation, and DNA methylation	Sustained stress response, amygdala hyperactivity, mood disorders
FKBP5	Glucocorticoid receptor co-chaperone, stress resilience	DNA methylation	Altered stress resilience, increased susceptibility to depression and PTSD
OXTR	Oxytocin receptor, social support buffering	DNA methylation	Modulated social stress buffering, impacts mental health
SLC6A4	Serotonin transport, mood regulation	DNA methylation, histone methylation (H3K4, H3K27)	Dysregulated serotonin signalling, increased anxiety and depression risk
COMT	Catecholamine metabolism, stress response	DNA methylation	Altered stress response, linked to psychiatric disorders
CRHR1	Corticotropin-releasing hormone receptor, stress response	DNA methylation	Dysregulated HPA axis, increased stress-related psychiatric risk
c-Fos	Synaptic plasticity, memory consolidation	DNA methylation, histone acetylation	Impaired memory formation, cognitive deficits under chronic stress
Arc	Synaptic plasticity, long-term potentiation	DNA methylation, histone acetylation	Reduced synaptic plasticity, cognitive impairments
EGR1	Emotional memory regulation, neural plasticity	DNA methylation	Increased amygdala reactivity, mood disorders
FosB	Neural plasticity, stress adaptation	DNA methylation	Altered stress response, linked to mood disorders
NR4A	Stress response, emotional regulation	DNA methylation	Dysregulated emotional processing, increased mood disorders
TNF	Pro-inflammatory response	DNA methylation	Enhanced inflammation, contributes to chronic diseases (*e.g*., cardiovascular, diabetes)
IL-6	Inflammatory signaling	DNA methylation	Chronic inflammation, increased risk of metabolic and cardiovascular diseases
TP53	Tumor suppressor, DNA repair, cell cycle control	DNA methylation (hypermethylation)	Increased cancer risk due to impaired DNA repair and cell cycle regulation
BRCA1	DNA repair, tumor suppression	DNA methylation	Increased breast/ovarian cancer risk due to impaired DNA damage response
RB1	Cell cycle regulation, tumor suppression	Histone deacetylation	Uncontrolled cell proliferation, increased cancer risk
LEP	Appetite control, metabolism	Histone acetylation	Dysregulated appetite, contributes to obesity
EDN1	Blood pressure regulation, vasoconstriction	Histone acetylation, methylation	Increased hypertension and cardiovascular risk
CYP1A1	Detoxification pathways	DNA methylation	Disrupted detoxification, increased susceptibility to environmental toxins
p16INK4a	Tumor suppression, cell cycle regulation	DNA methylation	Increased lung cancer risk due to impaired cell cycle control
eNOS	Cardiovascular health, nitric oxide production	DNA methylation	Impaired vascular function, increased cardiovascular disease risk
HOTAIR (lncRNA)	Chromatin remodelling, stress response regulation	DNA methylation	Altered stress-responsive gene expression, increased disease susceptibility
miR-132	Chromatin accessibility, stress response regulation	Targets histone acetyltransferases (CBP, p300, PCAF) and deacetylases (HDAC1, HDAC2)	Modulates neural plasticity, stress adaptation, and mood regulation
miR-21	Cell proliferation, apoptosis regulation	Downregulation under stress	Increased cancer risk, reduced apoptosis
miR-34a	Tumor suppression, colorectal cancer regulation	Downregulation under stress	Increased colorectal cancer risk due to impaired tumor suppression
miR-155	Immune function, inflammation regulation	Upregulation under stress	Enhanced pro-inflammatory state, increased chronic disease risk
miR-663	Anxiety regulation	Upregulation under stress	Increased anxiety symptoms
miR-4505	Anxiety regulation	Upregulation under stress	Correlated with anxiety symptoms
miR-29c	Social anxiety, prefrontal cortex connectivity	Upregulation under stress	Increased social anxiety, altered brain connectivity

**Table 2:** Summary of DNA methylation inhibitors with their mode of action.

**DNA Methylation Inhibitor**	**Mechanism of Action**	**Targeted Genes/Pathways**	**References**
5-Azacytidine	Nucleoside analogue that incorporates into DNA during replication, trapping and depleting DNA methyltransferases (DNMTs), leading to DNA hypomethylation and gene reactivation.	Various stress-responsive genes, *e.g*., NR3C1, BDNF, *etc*.	[190, 191]
Decitabine	Nucleoside analogue that functions similarly to 5-Azacytidine, incorporating into DNA during replication and inhibiting DNMTs, resulting in DNA hypomethylation and gene reactivation.	Specific genes involved in stress regulation, *e.g*., NR3C1, BDNF, *etc*.	[192, 193]
RG108	Direct inhibitor of DNMTs, covalently binding to these enzymes and inhibiting their catalytic activity, ultimately promoting DNA demethylation and potential gene reactivation.	DNMTs influence DNA methylation patterns globally.	[194, 195]
Epigallocatechin gallate (EGCG)	Functions as a DNA methyltransferase inhibitor by influencing DNMTs, promoting demethylation of specific genes and pathways involved in stress regulation.	Genes: NR3C1, BDNF, Fkbp5, COMT, HTR2A, OXTR, and Pathways related to stress adaptation.	[196, 197]
Zebularine	A cytidine analogue that may influence DNA methylation patterns at specific genomic loci, impacting stress responses.	Genes: NR3C1, BDNF, FKBP5, CRH, and Pathways related to stress adaptation.	[198, 199]
S-adenosylmethionine (SAMe)	A methyl donor, SAMe, indirectly influences DNA methylation by modulating methylation reactions and interacting with methyltransferases.	Genes: COMT, MAOA, BDNF, NR3C1, and Pathways related to stress adaptation.	[200, 201]
Curcumin	Acts as a DNA methyltransferase inhibitor, potentially inhibiting DNMTs and affecting the methylation status of specific genes involved in stress regulation.	Genes: BDNF, NR3C1, FKBP5, CRH, and Pathways related to stress adaptation.	[202, 203]
Valproic Acid	Simultaneously functions as a histone deacetylase (HDAC) inhibitor and DNMT inhibitor, impacting DNA methylation and histone modifications, thereby influencing gene expression related to stress.	Genes: NR3C1, BDNF, FKBP5, CRH, and Pathways related to stress adaptation.	[204, 205]

## LIST OF ABBREVIATIONS

BDNF: Brain-Derived Neurotrophic Factor
CGRP: Calcitonin Gene-Related Peptide
Gres: Glucocorticoid Response Elements
Hdacis: Histone Deacetylase Inhibitors
HPA: Hypothalamic-Pituitary-Adrenal
IL-6: Interleukin 6
IL-8: Interleukin-8
MA: Migraine With Aura
Ncrnas: Non-Coding Rnas
NR3C1: Glucocorticoid Receptor Gene
REM: Rapid Eye Movement
TNF-Α: Tumor Necrosis Factor-Alpha
